# Design of real-time transmission system for underwater panoramic camera based on RTSP

**DOI:** 10.1371/journal.pone.0320000

**Published:** 2025-03-26

**Authors:** Wenhui Wang, Yongqi Li, Rufei He

**Affiliations:** CSG PGC Power Storage Research Institute, Guangzhou, China; Jaramogi Oginga Odinga University of Science and Technology, KENYA

## Abstract

The underwater environment is complex and variable, with limited transmission bandwidth and strong clutter and interference signals, which make the collected images susceptible to adverse effects such as attenuation and diffusion, resulting in image signal transmission congestion. In the current transmission system application process, the interference noise present in the collected original image will increase the average reprojection error of the image; And some systems have complex data collection processes, requiring the deployment of multiple sensors, which increases data transmission time. In order to improve the ability and efficiency of underwater data collection and transmission, and ensure the quality of underwater multimedia data transmission, a real-time transmission system for underwater panoramic cameras based on RTSP (Real Time Streaming Protocol) was designed. In the data acquisition layer, after setting the relevant parameters of the underwater panoramic camera through camera calibration method, the calibrated underwater panoramic camera is used to collect underwater data. The collected underwater data is compressed and encoded by the encoder in the data encoding layer, and then transmitted to the RTSP server layer. After receiving compressed and encoded underwater data, the RTSP server layer stores it on disk and encapsulates it into RTP (Real-time Transport Protocol) packets. The congestion control method using RTCP feedback monitors the network status in real-time and dynamically adjusts the data transmission rate, effectively avoiding the occurrence of network congestion problems. The RTSP application layer client receives data from the RTSP server, implements restructuring analysis through RTP packets, and sends the restructured frame data into a double buffer waiting for decoding and playback or file recording, achieving real-time transmission of underwater panoramic cameras. The experimental results show that the data transmission effect and quality of the system are good, and the average reprojection error of the calibrated image is 0.12 pixels; During the process of data collection and transmission, the effective time window of the eye diagram is the largest, and the waveform of the top-level data transmission tends to be stable with the smallest fluctuation amsplitude; Dynamically adjust the data transmission rate, control the network packet loss rate below 0.5%, and improve the real-time transmission efficiency of underwater data.

## 1 Introduction

With the development of science and technology, the development and utilization of marine resources by humans have been very rapid, and some marine resource development technologies have gradually matured. With the continuous exploration of the underwater environment by humans, more and more marine resources are gradually being discovered. Marine resources are gradually demonstrating significant importance for human survival and development. However, due to the complexity of the marine environment for humans, a portion of marine resources exist in locations that humans cannot reach or explore for a long time. Therefore, the complex environment of the ocean hinders human exploration of the ocean and the development of marine resources [1]. In modern ocean development, more and more high-tech applications are being applied to traditional ocean development industries, leading to industrial upgrading. The application of information technology in ocean development will also promote the development and establishment of new ocean industries [2]. At present, the development and application of new materials, robots, ship technology, and resource protection and information collection technology in marine fisheries have led to significant changes in the field of marine development technology. The marine oil and gas industry, as an emerging marine high-tech industry, is also rapidly developing and maturing. However, due to the special characteristics of seawater, some marine equipment operating on the seabed, such as oil and gas exploration equipment [3], oil and gas transportation pipelines, equipment chains, and other development equipment, are very susceptible to corrosion by seawater. In addition, due to the extremely dangerous underwater environment for humans, marine equipment still needs to be transported to land for maintenance, resulting in resource waste. Therefore, some high-tech technologies are gradually applied to the detection of marine equipment, such as laser detection, monocular vision measurement, laser 3D scanning, structured light 3D reconstruction, binocular and multi camera 3D reconstruction technologies. These methods can detect underwater objects without human contact with underwater equipment. Both monocular visual measurement technology and binocular 3D reconstruction technology are non-contact systems, which are based on sensor design to capture object images and then process them to obtain relevant information [4]. They do not require human deep-sea operations, so they are gradually being widely used in underwater equipment monitoring. As an important tool in ocean exploration technology, underwater panoramic cameras are gradually being used to observe the dynamic habitat environment of underwater organisms [5], visual observation information, including shallow coral reefs, nearshore mangroves, and docks. Real time collection of underwater data through panoramic cameras [6], can generate streaming videos and images. Built-in image enhancement algorithm is used to improve the image quality of underwater images [7], and transmit them to client browsers for real-time viewing through data transmission networks. The real-time transmission rate of underwater panoramic cameras directly affects the interaction effect with clients, so relevant scholars have begun to study the real-time transmission system of underwater panoramic cameras.

Streaming media technology is a new technology used for multimedia information transmission and processing in network, and it has become the main solution for online video and audio transmission. In order to achieve high-quality data transmission within limited underwater bandwidth, a real-time transmission system for underwater panoramic cameras based on RTSP is designed. The rest of this article is organized as follows: Section 2 presents the related works, while section 3 describes the real-time transmission system for underwater panoramic camera based on RTSP. On the other hand, Section 4 provides the experimental analysis. Finally, Section 5 concludes this paper and offers some insights about future research directions

The main research contributions of this paper are as follows:

① The design system uses RTCP feedback mechanism to monitor network conditions in real-time, including parameters such as loop back time and packet loss event occurrence rate. These feedback information are used to dynamically adjust the sending rate to adapt to network changes, ensuring stable data transmission even when the network is unstable. This mechanism is based on RTCP feedback and adjusts the sending rate in real time according to the degree of network congestion, which helps to reduce data loss and transmission delay when the network is unstable, reduce resource consumption, and improve the reliability and stability of data transmission.
② The use of NW901 video encoding chip for efficient compression encoding of collected data not only improves data transmission efficiency and quality, but also helps reduce the risk of data loss.
③ The system adopts high-performance underwater panoramic cameras, video decoding chips, DMA controllers, SDRAM, NW901 video encoding chips, and DM8168 Da Vinci digital media processors and other high-performance hardware, providing strong hardware support for data transmission and improving the robustness of the design system.

This paper designs and experimentally verifies a real-time transmission system for underwater panoramic cameras based on RTSP, covering data acquisition, encoding, server processing, and client reception, effectively improving the quality and efficiency of underwater data transmission.

## 2 Related works

Reference [8] designed a data transmission system based on DDR2 (Double-Data-RateTwo), which adopted a hierarchical caching mechanism of internal FIFO (First In First Out) resources in FPGA (Field-Programmable Gate Array) combined with off chip DDR2. In order to facilitate image data reading, writing, and address management, the internal storage space of DDR2 was redistributed to complete data transmission. However, during the application process of the system, the data transmission accuracy is insufficient due to the interference noise existing in the collected original images. Reference [9] designed a data transmission system based on LoRa, which was a star network composed of LoRa wireless transmission technology. It could provide users with a simple data transmission system that could achieve long-distance and easy expansion. Among them, the LoRa terminal node was embedded with the Contiki embedded dedicated operating system, effectively reducing the memory consumption of user-defined multithreading and multitasking. The node adopted cyclic queue buffered data communication, which had the function of encapsulation into frames and periodic sleep. Heterogeneous gateways were responsible for storing and forwarding, and support various communication technologies such as NB IoT and Wi Fi to upload to remote cloud platforms. Terminal node information could be configured and displayed on the remote cloud platform. The system has a high design cost and low adaptability. Reference [10] designed a laser echo data acquisition and transmission system based on FPGA. The system used ADC (Analog to digital converter) and FPGA as the control core of the data acquisition system. In this system, ADC sampled the full waveform of weak echo signals and performed fast digital processing. FPGA performed logical control on the entire system and FIFO caching and accumulation averaging processing on the digitized data to achieve data transmission. The collection process of this system is too complex and requires the deployment of multiple sensors, resulting in poor application performance. Reference [11] designed a visible light character transmission system based on a microcontroller. The system used a line of sight link, light amplitude modulation direct detection technology to modulate the light emitting diode (LED) at high speed, and tested the system’s ability to resist environmental noise to achieve data transmission. The system requires a large amount of computation during data processing, resulting in poor data transmission timeliness. Reference [12] designed a wireless data transmission system for high-frequency ground wave radar, which was a radar data wireless transmission scheme. At the same time, a rapid evaluation method for the communication quality of the wireless transmission system was designed. The scheme established a wireless transmission link network through a wireless bridge and used TCP (Transmission Control Protocol) protocol to achieve reliable data transmission. Although the system performs well in a single scenario, it has poor performance in data collection and transmission in complex scenarios.

Compared with the above methods, this paper’s method receives compressed and encoded underwater data through the RTSP server layer, and uses the congestion control method feedback from RTCP to monitor the network status in real time and dynamically adjust the data transmission rate. Ensured real-time data transmission and effectively avoided network congestion. And through reasonable camera calibration and data encoding, the high quality of underwater data acquisition is ensured, and efficient data transmission is achieved through RTSP protocol. Through reasonable system design and efficient transmission protocols, high-quality real-time transmission of underwater multimedia data has been achieved while maintaining low system complexity and cost, achieving real-time transmission of underwater images.

## 3 Real-time transmission system for underwater panoramic camera based on RTSP

This system is a real-time transmission system for underwater panoramic cameras based on RTSP, aiming to achieve high-quality underwater video data transmission.

Step1: The core hardware is a high-performance underwater panoramic camera. The data collected by the camera is processed by a video decoding chip, cached in FIFO, and then transferred to SDRAM through a DMA controller.
Step2: Using the NW901 video encoding chip, the collected data is efficiently compressed and encoded to improve data transmission efficiency and quality [13,14].
Step3: Utilize the high-performance DM8168 Da Vinci digital media processor to ensure stable and fast data transmission to RTSP servers. The RTSP server software is responsible for receiving encoded data, performing RTP packaging, and pushing it to the network. Meanwhile, by dynamically adjusting the sending rate through the RTCP feedback mechanism, network congestion control is achieved.
Step4: Adopting TCP friendly congestion control mechanism (TFRC), based on RTCP feedback, the sending rate is dynamically adjusted according to parameters such as loop back time and packet loss event occurrence rate.
Step5: The client receives RTP packets, performs reassembly analysis, and sends them to a double buffer for decoding, playback, or file recording.

Through the above design, the system can transmit high-quality underwater panoramic video streams in real-time and efficiently [15,16], while ensuring the stability and reliability of network transmission.

### 3.1 Overall system structure

**Fig 1:**
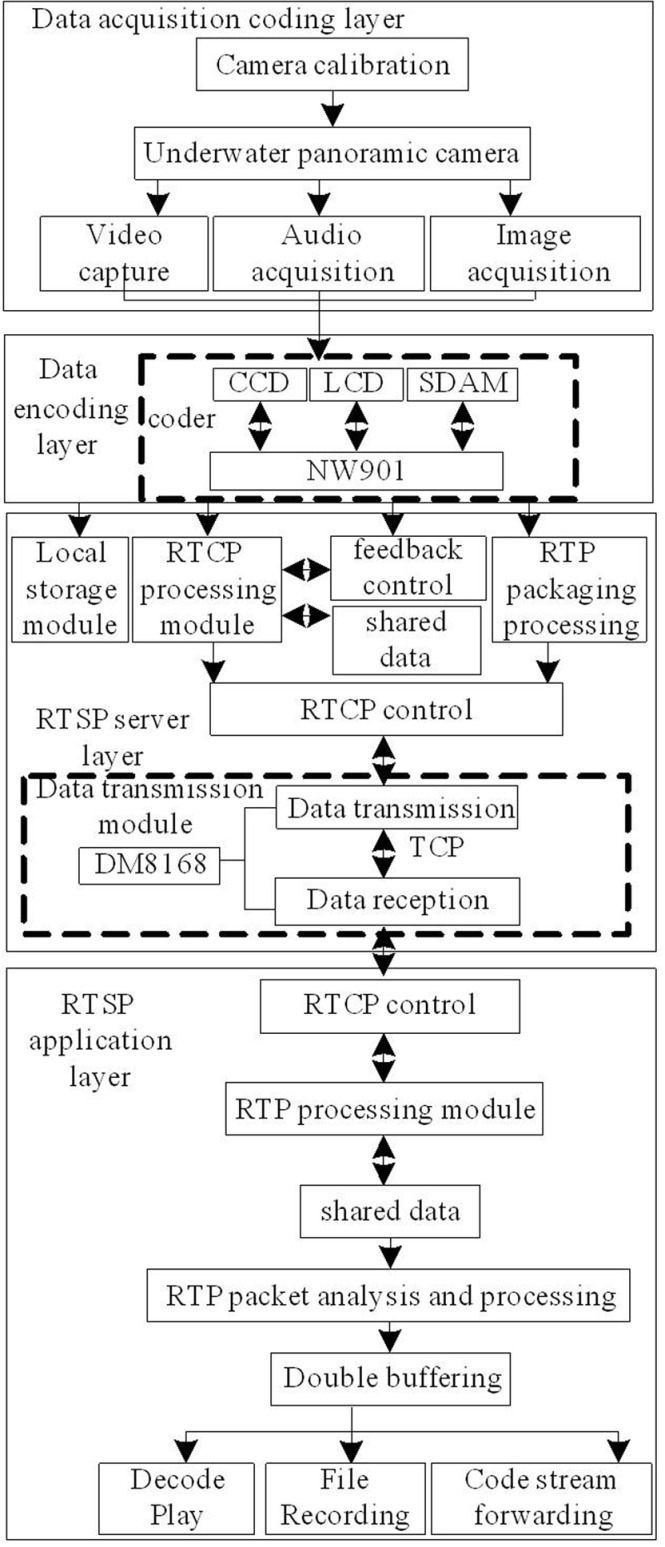
Overall System Structure.

The RTCP protocol is mainly used to transmit control information between session members participating in multimedia data communication. In the design system, RTCP is mainly used to provide feedback information on network congestion and dynamically adjust the data transmission rate accordingly. Considering the strong ability of RTSP to transmit control information and its good application effect in data transmission control, a real-time transmission system for underwater panoramic cameras based on RTSP is designed. The overall structure of the system is shown in Fig 1.

As shown in Fig 1, the system mainly consists of a data acquisition layer, a data encoding layer, an RTSP server layer, an RTSP application layer, and so on. After setting the position of the underwater panoramic camera reasonably through camera calibration method in the data acquisition layer, the calibrated underwater panoramic camera is used to collect data [17,18]. The collected data is compressed and encoded by the encoder in the data encoding layer and transmitted to the RTSP server. After receiving the compressed and encoded data, the RTSP server saves it on disk and encapsulates it into an RTP packet, which is sent to the RTSP application layer. The RTSP application layer client receives the RTP packet for reassembly analysis, and then sends the reassembled frame data to the double buffer for decoding, playback, or file recording. Verified by the RTCP feedback control module, using RTCP feedback to complete RTSP interaction can effectively control network congestion. The main function of the RTCP processing module is to provide and process feedback information, because while the client receives and processes RTP packets, it will calculate the current RTP packet reception status (such as interval jitter and packet loss rate), and then put the statistical information into the shared information area. When the next RTCP interval arrives, the receiver report RR packet will be sent, and the server can change the bitstream transmission rate accordingly based on this feedback information. This can effectively improve network congestion and fully utilize network resources.

### 3.2 Design of data acquisition layer

#### 3.2.1 Data acquisition layer hardware.

**Fig 2:**
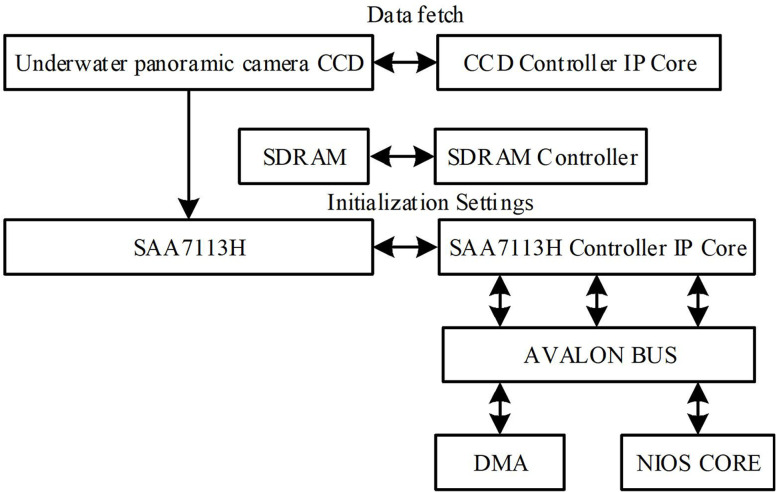
Hardware architecture of data acquisition layer.

The data acquisition layer is a key component of the entire system, responsible for capturing real-time video, audio, and image data from underwater environments. The core hardware device of this layer is the underwater panoramic camera, which can not only capture vast underwater scenes but also ensure real-time and accurate data. The core hardware of the data acquisition layer is an underwater panoramic camera, which collects real-time data such as underwater video, audio, and images through the underwater panoramic camera. The hardware architecture of the data acquisition layer is shown in Fig 2.

As shown in Fig 2, after the system is powered on, the CCD_ Controller submodule and the SAA7113H_Controller initializes the camera submodule of the underwater panoramic camera and the SAA7113H video decoding chip respectively. The underwater panoramic camera captures video data [19], which is processed by the SAA7113H video decoding chip and quickly calculated into the FIFO for data caching processing. Then, the DMA controller directly transfers the data from the FIFO to the SDRAM memory. The CPU and DMA controller respectively access SDRAM through Avalon bus to complete video signal reading and writing operations.

#### 3.2.2 Camera calibration method.

Camera calibration is an important task in underwater vision systems, directly related to the accuracy of target localization and underwater measurement. Due to the unique nature of underwater environments, light undergoes refraction during propagation, resulting in changes in the camera imaging model. Therefore, accurate calibration of underwater cameras is particularly important. Underwater camera calibration is the foundation for achieving underwater target positioning and measurement [20]. A complete refractive camera model is established based on the propagation process of light in water, waterproof shell, and air. Then, the refractive camera model is calibrated. Due to the refractive effect of water, light rays will be refracted when entering the underwater camera, causing changes in the camera imaging model. Based on this, the internal and external parameters of the camera are calibrated using the Zhang Zhengyou calibration method. This calibration method extracts feature points such as checkerboard corners through image processing techniques, and establishes corresponding relationships using the coordinates of these feature points in the image and the world coordinate system. Through this correspondence, the internal parameters of the camera (such as focal length, principal point coordinates, distortion coefficient, etc.) and external parameters (such as rotation matrix, translation vector, etc.) can be solved. For the shell parameters, the error model between the corresponding points of space points on the imaging plane and the actual imaging plane points is established, and the particle swarm optimization is used to solve it.

In the underwater refraction camera model, the parameters that need to be calibrated mainly include the internal parameters of the camera, the external parameters of the camera, and the waterproof shell parameters. Among them, the waterproof shell parameters mainly include the distance *a* from the optical center to the refractive plane of the air-waterproof shell, the thickness *l* of the waterproof shell, and the normal vector $b_{\pi }\left(b_{x},b_{y},b_{z}\right)$ of the refractive plane.

The calibration method for camera basic parameters is as follows:

The camera’s internal parameters include focal length *f*, principal point coordinate $\left(u_{0},v_{0}\right)$, and distortion coefficient matrix $g_{c}$; The external parameters of the camera include the rotation matrix *R* and the translation vector *H*. Due to the fact that the internal and external parameters of the camera do not change with the environment, it is possible to calibrate the camera parameters on land first. The commonly used camera calibration method at present is the Zhang Zhengyou calibration method [21–23], which establishes the corresponding relationship between the checkerboard points and the imaging plane points based on the images of multiple flat checkerboard calibration boards with different angles. The calibration idea is to first detect feature points from the image, then solve for the internal and external parameters of the camera under ideal distortion-free conditions, and use maximum likelihood estimation to improve accuracy. Then, it uses the least squares method to solve for distortion parameters, and finally uses maximum likelihood estimation for optimization to ultimately obtain the camera parameters.

According to the analysis of the underwater refractive camera model, there are three shell parameters that need to be calibrated: The normal vector $b_{\pi }$ of the refractive plane, the distance *a* from the optical center to the refractive plane of the air-waterproof shell, and the thickness *l* of the waterproof shell. An error model between the corresponding point *q*^′^ on the imaging plane and the actual imaging plane point *q* based on the spatial point *q* solved by the model is established, as shown in formula (1):


$$F=\frac{\min\left\|q_{i}-{q^{\prime }}_{i}\left(b,a,l\right)\right\|}{n}$$ (1)

In formula (1), ${q^{\prime }}_{i}\left(b,a,l\right)$ represents the coordinates of the *i* -th feature point in the *j* -th image corresponding to the point on the imaging plane solved by the model; $q_{i}$ represents the coordinates of the *i* -th feature point in the actual imaging plane of the *j* -th image; *n* represents the number of feature points in the *j* -th image.

The error model is a multi-parameter optimization problem, which is processed by particle swarm optimization [24]. This algorithm simulates birds in a flock of birds by designing particles, which have two attributes: speed and position. At the beginning, the particles are initialized, allowing each particle to search for the optimal solution and share it with other particles to obtain the optimal solution as the current local optimal solution. All particles adjust their position and velocity based on their own solution and the current local optimal solution, iteratively calculating until the termination condition is reached, in order to obtain the global optimal solution. In the error model, there are five parameters that need to be solved, namely the distance *a* from the optical center to the refractive plane of the air waterproof shell, the thickness *l* of the waterproof shell, and the normal vector $b_{\pi }\left(b_{x},b_{y},b_{z}\right)$ of the refractive plane.

The process of solving by particle swarm optimization [25-27] is as follows:

(1) Initialize. The initial population size is the total number of characteristic points. The position $\chi_{i}$ and velocity $\nu_{i}$ of each parameter are initialized randomly. The initial fitness $P_{i}$ of each particle is set as the individual extreme value $pBest_{i}$, and the optimal $\min\left(pBest_{i}\right)$ of all individual extreme values is set as the global extreme value gBest.
(2) Update extreme values. The current fitness $P_{i}$ of the particle is compared with the historical extreme value $pBest_{i}$. If the current fitness $P_{i}$ is better, update the historical extreme value $pBest_{i}$ with the current fitness $P_{i}$, and compare it with the global extreme value gBest. If the current fitness P is better, update the global extreme value gBest with the current fitness $P_{i}$.
(3) Update location and speed.

Update the velocity $\nu_{i}$ of the particle, which is expressed as follows:


$$\nu_{i}\left(\lambda +1\right)=Fc_{1}\beta_{1}\left(pBset_{i}\left(\lambda \right)-\chi_{i}\left(\lambda \right)\right)+c_{2}\beta_{2}\left(gBset_{i}\left(\lambda \right)-\chi_{i}\left(\lambda \right)\right)+\nu_{i}\left(\lambda \right)$$ (2)

Update the position $\chi_{i}$ of the particle, and its expression is as follows:


$$\chi_{i}\left(\lambda +1\right)=\nu_{i}\left(\lambda +1\right)+\chi_{i}\left(\lambda \right)$$ (3)

Where, the inertia weight coefficients are represented by $\beta_{1}$ and $\beta_{2}$, and the learning factors are represented by $\beta_{1}$ and $c_{2}$, respectively.

(4) Judge the termination condition. The termination condition is that the current fitness of the particle is less than the predetermined fitness threshold or the iterative calculation reaches the predetermined number of times. If the termination condition is reached, find the optimal solution, otherwise continue to calculate the fitness and update the extreme value.

After the above optimization process, the parameter values under the optimal solution conditions are finally obtained, which are the calibration values of the waterproof shell parameters.

### 3.3 Design of data encoding layer

**Fig 3:**
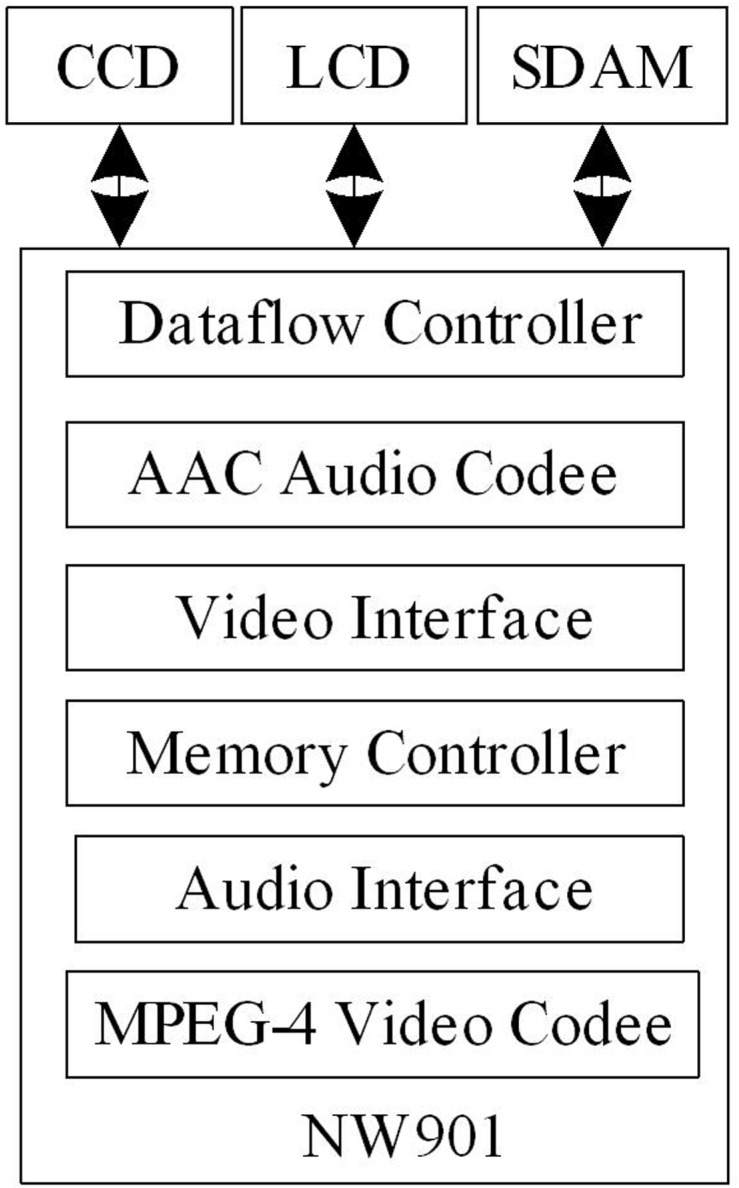
Structure of NW901 video encoding chip.

The data encoding layer is a key component in underwater vision systems, responsible for compressing and encoding the raw data collected by underwater panoramic cameras for data transmission and storage. The design of this layer directly affects the efficiency of data transmission and the quality of video images. The data collected by the underwater panoramic camera are transfered to the encoder module, compress and encode the collected data by calling the encoding function, and wait for the RTSP server to call. The core hardware of the encoder module is the NW901 video encoding chip, which is small in size and highly integrated. The video encoding chip has high encoding efficiency and quality, and has advanced encoding algorithms that can provide high-quality video images; This chip has the advantages of programmability, flexible host and peripheral interfaces to adapt to the dynamically changing market demand. NW901 is a specialized video encoding chip produced by DIVIO company. The video encoding and decoding adopts the advanced and simple level of MPEG-4 standard, which can provide motion images with a frame rate of up to 30fps similar to television quality effects. The structure of the NW901 video encoding chi is shown in Fig 3.

As shown in Fig 3, the NW901 video encoding chip mainly includes hardware such as a data flow controller, AAC audio encoding, and video interface. NW901 will compress and encode the collected encoded data to improve data transmission efficiency. Simultaneously, CCD, LCD, and SDAM perform data exchange. The main features of NW901 include:

(1) Video encoding. The image format is SIF/CIF, with a frame rate of up to 30 fps, adopting a 1/4 pixel motion estimation algorithm, which is capable of scene change detection and rate control. A preprocessor is used to reduce time-domain signal-to-noise, equipped with deblocking and dering filters. The transmission speed is from 64 kbps to 2 Mbps, with prediction of AC and DC coefficients.
(2) Audio encoding. It adopts AAC encoding technology, providing high-quality dual channel stereo sound; Provides eight selectable bitrates of 16, 24, 32, 48, 64, 96128256Kbps.
(3) Video capture device. The four megapixel camera has automatic exposure and automatic white balance functions; JPEG compression rate optional; Pixel interpolation repair function; Automatic flash control and lens zoom control.
(4) Audio and video input/output interface. In addition to sensor input, the chip has the ability to process and output digital video according to the ITU-456 standard, and the digital audio input/output uses the I2S interface.
(5) Host interface. The interface of NW901 is designed to be compatible with various sensors and LCDs. Therefore, the chip has a set of large capacity registers to set decoding parameters such as video format, bit rate, and spacing between I and P frames to meet specific sensor and LCD latency and control needs.

### 3.4 Design of RTSP server layer

#### 3.4.1 Design of data transmission module.

**Fig 4:**
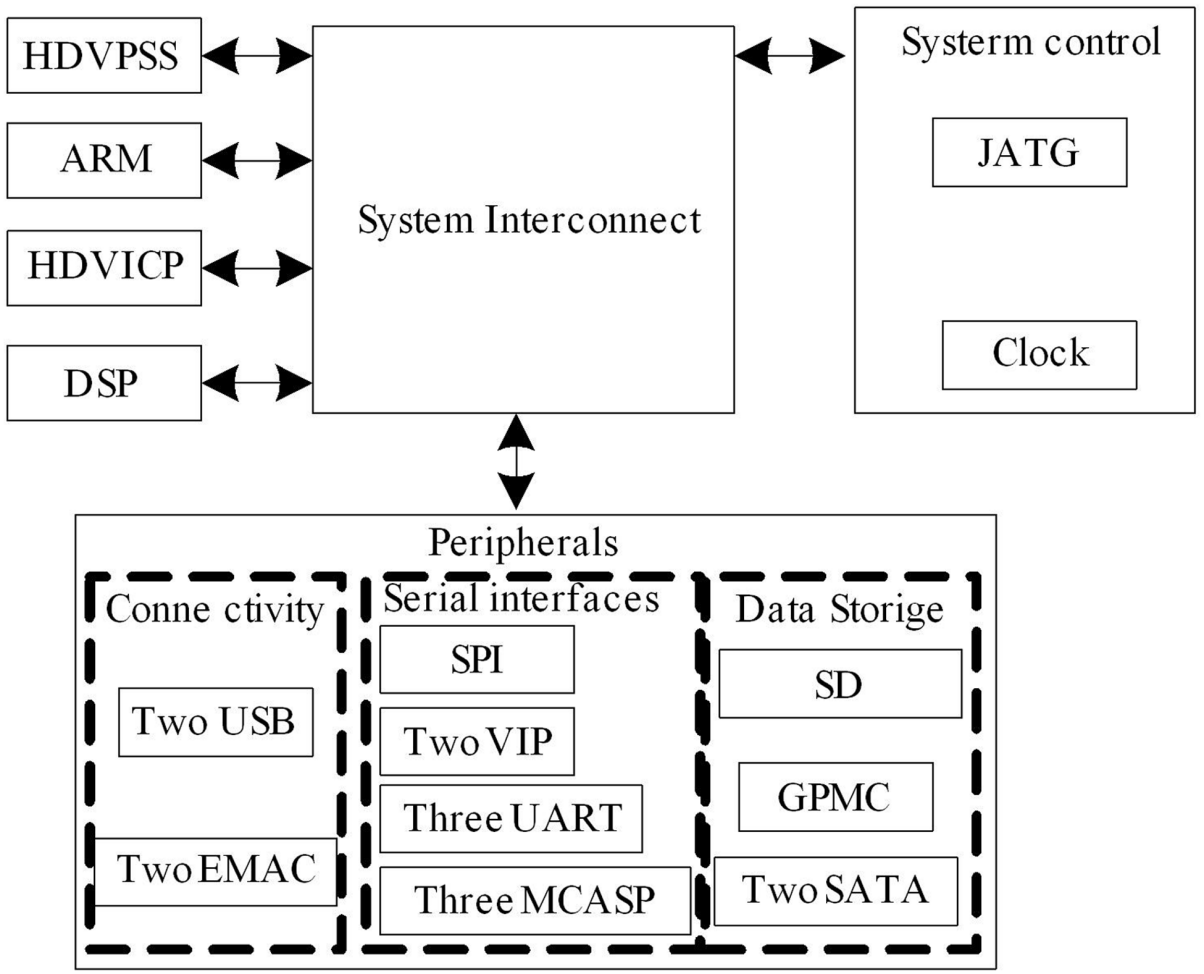
Overall structure of DM8168 Da Vinci digital media processor.

The data transmission module is an important component of the RTSP server layer, responsible for efficiently transmitting encoded data to the network. In this module, the high-performance DM8168 da Vinci digital media processor is used, which can process multiple high-definition video streams at the same time. It is an ideal choice for high-definition video monitoring, video conferencing and other applications. The data transmission module serves as a data exchange channel and adopts a high-performance DM8168 da Vinci digital media processor. The launch of DM8168 further strengthens the product camp of the TI da Vinci digital media processor platform. This product highly integrates a 1.2 GHz ARM Cortex-A8 and a 1 GHz C674x DSP chip as well as a video graphics accelerator on the chip. It can simultaneously process three 1080p60 fps video streams, achieving real video communication. It is an ideal choice for high-definition video monitoring systems, video conferencing systems, and video broadcasting systems. The overall structure of the DM8168 da Vinci digital media processor is shown in Fig 4.

As shown in Fig 4, DM8168 has a rich set of peripheral devices, which not only controls external devices but also enables communication with external processors. The peripheral set of the DM8168 development board used in this system includes a high-definition video processing submodule (HDVPSS), which mainly controls the collection, display, and denoising of videos. It supports the input and output of two HDMI high-definition videos, or the synchronous input and output of multiple high-definition and standard definition videos; 2 video interface processors (VIPs) and 3 audio access interfaces (MCASPs) are responsible for audio and video input; 3 asynchronous transceiver (UARTs) for communication with auxiliary equipment; SPI port, which can carry a WIFI module on board; 2 Gigabit Ethernet ports and 2 USB ports, expandable as 3G and GPS wireless communication modules; In terms of storage, it includes 8 pieces of 2G DDR3 memory, 2 SATA interfaces for external mobile hard drives, and 1 SD card port. The core of the ARM submodule is the ARM CortexTM-A8 RISCPU, with a main frequency of up to 1.2 GHz and a cache of 32K bytes (KB) of instructions; 32 KB data cache; 256 KB L2 cache; 48 KB ROM and 64 KB RAM. The DSP sub module adopts the TI C674x VLIW floating point DSP core, with a main frequency of 1 GHz. Developers can transplant some video processing algorithms to this sub system, thereby reducing the system resources on ARM and reducing the complexity of the system software. HDVCIP is a high-definition video coprocessor [28]. The most crucial feature of this processor is that it has three HDVCIP2, each of which can independently encode or decode a single H.264 video with a resolution of up to 1080p60, or multiple lower resolution or frame rate videos. Encoding and decoding do not occupy DSP resources at all, which allows the system to complete higher complexity algorithms.

#### 3.4.2 Software design of RTSP server.

The RTSP server is the core part of the system, responsible for obtaining encoded data from the data encoding layer, performing RTP packaging processing, and then sending it to the network for reception by the client. In the software design of RTSP server, efficient algorithms and processes are adopted to ensure the real-time and integrity of data. In the system, the RTSP server is mainly responsible for obtaining encoded data from the data encoding layer, performing RTP packaging processing, and then sending it to the network through the data sending module in data transmission layer to wait for the client to receive it; Simultaneously creating an RTCP processing thread to dynamically calculate the current network sending rate by processing RR and SR packets, in order to achieve network congestion control. In addition, the server adopts the RTSP streaming media interaction protocol for session control of the RTSP application layer client. Firstly, an RTSP server is established. When a connection is established with the client, the server will promptly create an RTP session that responds to the client. The encoded frame data will be obtained from the front-end device for RTP encapsulation, which is to configure a 12 byte RTP message header according to the packet format of the RTP data transmission protocol, including version number, flag bit, sequence number, timestamp, and synchronization source information. Then the frame is cut into frame data smaller than the maximum transmission unit (MTU) of the network and loaded into the data load segment of the RTP message. The UDP or TCP header is encapsulated in the transport layer, and the IP header is encapsulated in the IP layer, and finally sent to the Internet.

**Fig 5:**
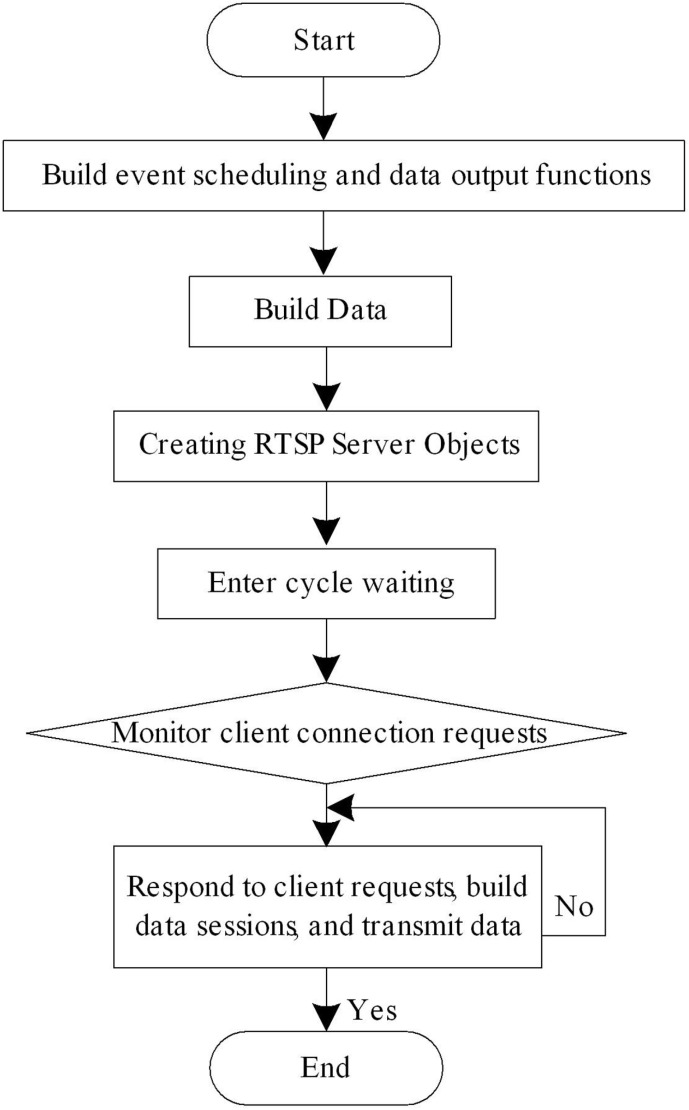
Basic design flow of RTSP server.

The RTSP server is the core part of the system, and its main function is to encapsulate and stream image data using RTSP. The main function of an RTSP server is to communicate between the client and server, build streaming data, and send RTSP streaming data. The basic design process of the RTSP server is shown in Fig 5.

As shown in Fig 5, when the RTSP server is running, it will first listen for the current TCP connection status. When it hears connection requests from the RTSP application layer client, the server will establish a new socket and bind the corresponding port number. Then the server enters the RTSP event loop, and after receiving the connection information sent by the client, it connects to the client. While waiting for the connection, the RTSP server opens up new threads to construct streaming media transmission data. After receiving data from the encoder, the RTSP server will construct an AVFrame type frame structure for subsequent streaming media push. The frame structure mainly includes image encoding data, encoder header file information, P frame, I frame, timestamp, etc. After successfully connecting to the client, the RTSP server obtains the streaming media ID by parsing the received information, establishes a streaming media session, and transmits streaming media data through the session. After the session is established, the system will create a data push address, establish a TCP connection, and build a dynamic listener to listen for return values from the client. If the return value is correct, RTSP push of image data will begin. After the push ends, the server closes the RTSP data push and removes the streaming session.

#### 3.4.3 Congestion control method based on RTCP feedback.

In streaming media transmission systems, network congestion is a common problem that directly affects the quality of media streams received by users. In order to effectively solve this problem, a congestion control method based on RTCP feedback was adopted. Congestion phenomenon refers to the phenomenon where the number of packets in a network is too large to be processed in time, resulting in a decrease in the performance of this part or even the entire network. In streaming media transmission systems, network congestion often occurs, which greatly affects the quality of media streams received by users. Congestion control, as an effective way to reduce network congestion, is of great significance in practical research. Due to the fact that User Datagram Protocol (UDP) itself does not have congestion control and error control mechanisms similar to TCP, packet loss and congestion phenomena are more severe in practical applications, and effective congestion control must be implemented [29–31]. Therefore, the congestion control mechanism based on RTCP feedback in the paper is designed for UDP mode. At present, UDP based end-to-end congestion control mechanisms can be divided into two categories: one is the Additive Increase Multiplicative Decrease (AIMD) mechanism that mimics TCP and the other is an improvement based on it; The other type is TCP Friendly Rate Control (TFRC), which is a mechanism for congestion control by adjusting the sending rate based on the TCP throughput model equation proposed by Padhye et al.

The first type of method mainly imitates AIMD congestion control of TCP. Due to the sudden and significant rate changes that the AIMD model may bring, multimedia senders must quickly adjust the sending rate based on TCP flows, which is not conducive to multimedia applications. On the one hand, it is difficult to find a suitable encoder in practical applications to achieve rapid and significant changes in output rate, and on the other hand, this rapid change can also make users feel uncomfortable.

The second type of method TFRC is a rate-based TCP friendly congestion control mechanism. It adjusts the bitstream sending rate based on complex TCP throughput formulas, mainly for unicast communication. The receiver periodically provides feedback to the server regarding the received data packets, and the sender calculates a new friendly rate based on the feedback information, thereby adjusting the current transmission rate accordingly. After startup, the sender immediately enters a slow start process similar to TCP, rapidly increasing the rate to the fair bandwidth value. When encountering the first loss event, the receiver ends the slow start process and then switches to a receiver based approach. In addition, TFRC does not aggressively seize bandwidth, but smoothly increases the transmission rate based on a decrease in lost event rates. It does not quickly reduce the transmission rate due to the loss of a single packet, but only halves the transmission rate after multiple consecutive packets are lost. Therefore, compared to the jitter factor caused by rapid rate changes in the AIMD mechanism, the stationarity of the TFRC mechanism makes it more suitable for use in streaming media transmission systems.

The TCP steady-state traffic formula is:


$$Z=W/\left\{U\sqrt{\frac{2_{\alpha \varepsilon }}{3}}+\eta_{RTO}\left[3\times \sqrt{\frac{3_{\alpha \varepsilon }}{8}\varepsilon \left(1+32\varepsilon^{2}\right)}\right]\right\}$$ (4)

In formula (4), *Z* is the average throughput rate, in bytes per second (B/s); *W* is the size of the message, in bytes (B); *U* is the link loopback time Round Trip Time (RTT), in seconds (s); $\eta_{RTO}$ is the retransmission timeout time of TCP, in seconds (s); *ε* is the packet loss event rate, ranging from 0 to 1.0; *α* is the number of received packets confirmed for each TCP response, defaulting to 1.

In the feedback control module of the RTSP server layer, the transmission rate is controlled through the RTCP feedback congestion control method. This method is mainly based on the rate-based TCP friendly congestion control mechanism. The receiver sends a RR packet to the server every RTCP interval, and the sender calculates the loopback time *U* and the packet loss event occurrence rate *ε* based on the received information of the RTP packet in the RR packet. Then, the TCP steady-state flow formula is imported, to calculate the average throughput rate that the current transmission should have, and effectively control the data transmission rate. Therefore, to calculate the average throughput rate, it is necessary to obtain parameters such as link loopback time *U*, retransmission timeout $\eta_{RTO}$, and packet loss event rate *ε*. Next, we will analyzed the process of obtaining these parameters and the mechanism for adjusting the transmission rate in detail.

(1) Obtaining parameters
(a) Loop back time *U*. Link loopback time refers to the period from the sender sending a sender report SR packet to the receipt of the latest receiver report RR packet. Each RTCP cycle has its own relevant sampling $U_{sample}$, and its calculation formula is as follows:

$$U_{Sample}=U_{NOW}-U_{LSR}-U_{DLSR}$$ (5)

Where, $U_{NOW}$ is the current time when the sending end received the RR packet, $U_{LSR}$ is the middle 32-bit value of the NTP timestamp in the most recent SR packet received by the receiving end, and $U_{DLSR}$ is the delay from the receiving end’s most recent SR packet to the sending end of the current RR packet. Both $U_{LSR}$ and $U_{DLSR}$ are stored in the received report block RR packet received by the source.

(b) Retransmission timeout $\eta_{RTO}$. The sending end will start a timer when sending data. If the receiving end fails to receive confirmation of the data segment within the specified time, the timer will timeout. The period from the start of the timer to the occurrence of the timeout is called the timeout time $\eta_{RTO}$ of the timer. The timeout time is generally longer than the link loopback time, otherwise redundant resend events will occur; But it cannot be too different from the loopback time, otherwise when a loss event occurs, the receiving end will not receive the retransmitted data segment for a long time, leading to greater transmission delay. The congestion control mechanism based on RTCP feedback in this paper is designed for UDP transmission, so there is no problem of timeout retransmission. Therefore, $\eta_{RTO}$ is generally set to 4 times the link loopback time, which is 4R.
(c) The occurrence rate of packet loss events is *ε*. To achieve TCP friendly RTCP feedback control, it is not feasible to only obtain the loss packet ratio in RR packets, as TCP mainly responds to packet loss events and cannot only consider the actual number of lost packets. Therefore, the loss of multiple consecutive packets within an RTCP interval is generally regarded as a loss event, and no matter how many packets are lost, the congestion window is only halved once.
(2) Adjustment of sender speed

The congestion control method based on RTCP feedback imitates the slow start mode of TCP to detect the available bandwidth of the network, doubling the transmission rate within each RTCP interval before packet loss occurs. When receiving a RR packet with a non-zero Fraction Loss threshold, it can calculate the various parameters analyzed above and substitute them into the TCP steady-state flow formula to calculate the current TCP friendly sending rate *Z*. Then it is compared with the current sending rate $Z_{Sample}$:

(a) If $Z_{Sample}<Z$, it indicates that the current network condition is good and there is excess link bandwidth. Therefore, within the next RTCP interval, the sender sets $Z_{Sample}=Z_{Sample}+\frac{1}{U}$.
(b) If $Z_{Sample}\geq Z$, it indicates that the current network is saturated, then within the next RTCP interval, *Z* will be used as the current data transmission rate, i.e. $Z_{Sample}=Z$.

Afterwards, whenever the sending end receives an RR packet, the TCP-friendly sending rate will be calculated by formula (4), and the data sending rate will be dynamically adjusted by comparing it with the current sending rate, thereby effectively controlling network congestion.

## 4 Experimental analysis

In order to verify the effectiveness of the real-time transmission system for underwater panoramic cameras based on RTSP, the rivers in the middle reaches of the Yellow River were taken as experimental objects. The river has a total length of 1345km and a drainage area of 219000 km^2^, of which mountains account for 35.7%, hills account for 23.5%, plains account for 34.5%, and sand dunes account for 6.3%. The terrain of the drainage basin is basically tilted from north to south and from east to west to the middle. The system designed in this paper is applied to the data transmission of the river, to test the application effect of the design system, and collect river ecological environment data for the river management department. The systems of reference [8], the systems of reference [9], the systems of reference [10], the systems of reference [11], and the systems of reference [12] are selected as experimental comparison systems for testing. The GoPro MAX model of 360 degree underwater panoramic camera is selected for underwater data collection. The GoPro MAX multifunctional dual lens camera has one lens in front and one lens in the back, allowing users to freely switch between shooting lenses. In addition to turning on dual lenses to capture panoramic images, users can also alternate between front and rear single lenses, integrating traditional GoPro videos and photos, panoramic imaging functions, and Vlog configuration. MAX has four shooting focus segments that can be switched on demand, and the newly added PowerPano extended panoramic function supports photo and video shooting. It also has 6 built-in microphones to capture 360 degree panoramic sound effects. This upgrade also includes camera built-in image stitching, allowing users to easily export and edit panoramic materials. The MAX body comes with a touch screen and built-in directional microphone, which has good waterproof performance.

### 4.1 Calibration test of underwater camera

The underwater camera calibration method is used to calibrate the internal and external parameters of the underwater panoramic camera in the air. 50 checkerboard calibration board photos are taken in the air using an underwater panoramic camera, and the posture of the calibration board is changed during the shooting process. The number of squares on the calibration board is 12 × 9. The side length is 30 mm. After obtaining the images, Zhang Zhengyou calibration method is used for calibration. During the calibration process, images with large errors are removed, and 10 calibration images are ultimately used. After calibration, the internal and external parameters of the underwater panoramic camera are obtained as shown in Table 1. The internal parameters of the underwater panoramic camera include focal length, principal point coordinates, distortion coefficient matrix, and the external parameters of the camera include rotation matrix and translation vector.

**Table 1:** Internal and external parameters of underwater panoramic camera.

Parameter	Short-cut process
Distortion coefficient	[-0.012,0.0002]
Rotation matrix	[0.9888 0.0018 -0.00118}
	[0.0018 0.9888 -0.00129}
	[0.0018 0.129 -0.9888}
Focal distance	[636.562,691.79]
Translation matrix	[-119.825,0.1492,0.4662]
Principal point	[-0.1598,-0.0038,0.0316]

There are three parameters for the waterproof shell of a panoramic camera: The distance *a* from the optical center to the refractive plane of the air-waterproof shell, the thickness *l* of the waterproof shell, and the normal vector $b_{\pi }\left(b_{x},b_{y},b_{z}\right)$ of the refractive plane. It is necessary to shoot the checkerboard calibration plate image, process and air in the water, obtain the underwater calibration plate image, extract the corner points in the image, and use particle swarm optimization to optimize and solve the shell parameters according to the error model. The results of the shell parameters are shown in Table 2.

**Table 2:** Parameters of underwater panoramic camera shell.

Parameter	Short-cut process
Refractive plane normal vectortor	[0.0226, -0.0395,0.09825]
Thickness of waterproof enclosure/mm	4.86
The distance from the optical center to the refractive plane of the air waterproof housing/mm	8.2659

**Fig 6:**
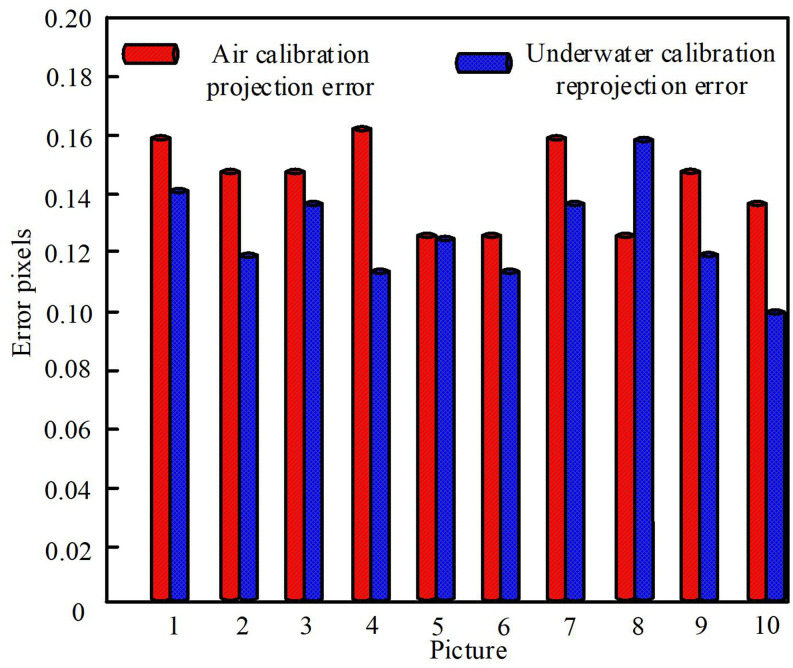
Reprojection error of calibration.

The calibration results are evaluated by using reprojection errors. According to the calibration parameters, it can calculate the corresponding point where the corner points in the chessboard calibration board image are projected onto the imaging plane, and compare them with the actual imaging points on the imaging plane to calculate the error between the two, which is the reprojection error. The average reprojection error of the calibration image is calculated, and the results are as shown in Fig 6.

The camera calibration method is used to obtain the optimal parameters of the underwater panoramic camera. In the calibration image, the average reprojection error of the air calibration image of underwater panoramic camera is 0.14 pixels, and the average reprojection error of the underwater calibration image of underwater panoramic camera is 0.12 pixels. This indicates that using the phase calibration method to calibrate the underwater panoramic camera can improve the accuracy of the underwater panoramic camera’s image acquisition and provide a data foundation for the system.

### 4.2 Data transmission effect testing

**Fig 7:**
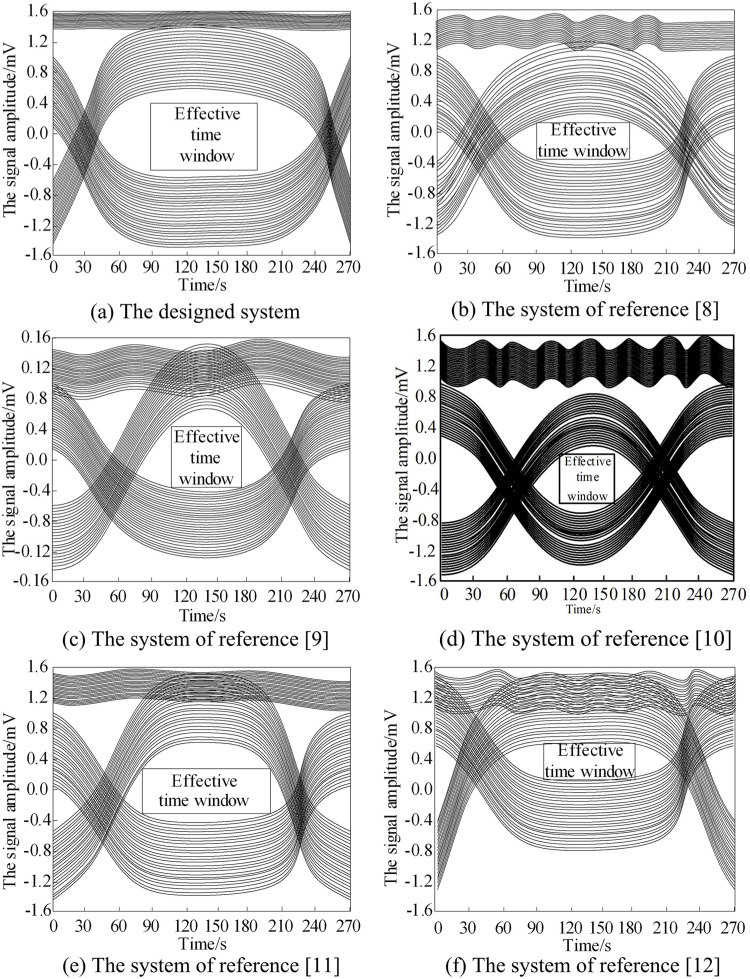
Data transmission effect test. (a) The designed system. (b) The system of reference [8]. (c) The system of reference [9]. (d) The system of reference [10]. (e) The system of reference [11]. (f) The system of reference [12].

The eye pattern of the collect data transmission waveform using the system designed in this paper and the systems of reference [8], the systems of reference [9], the systems of reference [10], the systems of reference [11], and the systems of reference [12] is made statistics to evaluate the data transmission effect of the designed system. The larger the effective time window of the eye pattern is, the better the data transmission effect is. The data transmission effect of the six systems is analyzed, and the analysis results are shown in Fig 7.

From Fig 7, it can be seen that the effective time window of the eye pattern in the design system during data collection and transmission is significantly larger than that of the other five systems during data collection and transmission. The effective time window of the eye pattern in the design system is the largest, and the fluctuation amplitude of the data transmission waveform at the top layer is the smallest, while the fluctuation amplitude of the data transmission waveform at the top layer of the other five systems is relatively large. Through comparative analysis, it can be concluded that the data transmission effect in this paper is the best.

**Fig 8:**
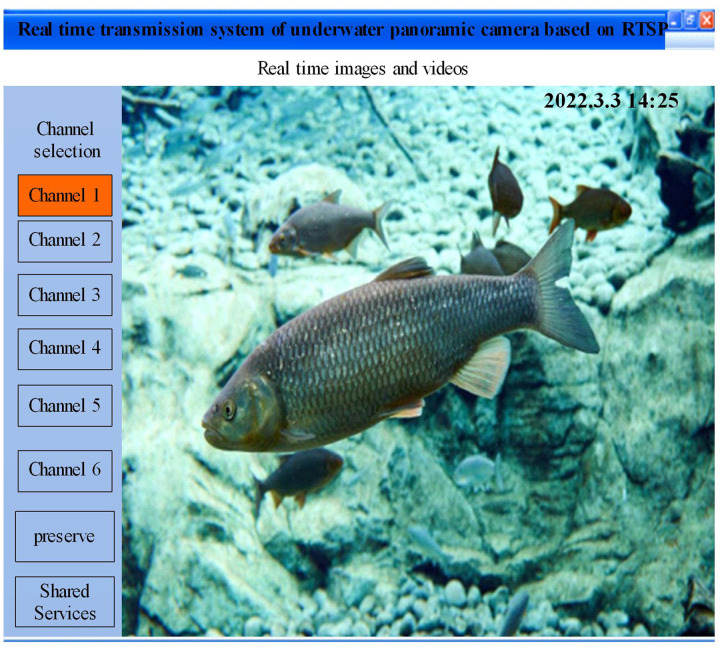
Real-time screen on the client.

To further verify the data transmission effect of the design system, the river’s hydrological management department uses the design system to detect fish in the river. River’s hydrological management personnel can view the real-time dynamics of fish in the river through the RTSP client, and the design system has a real-time screen on the client, as shown in Fig 8.

Analyzing Fig 8, it can be seen that river’s hydrological management personnel can view the dynamics of fish in the river in real-time through the RTSP client. The transmission image quality in the client is clear, and there is no lag phenomenon. This indicates that when using the RTCP feedback congestion control method in the design system, it can dynamically adjust the data transmission rate by comparing it with the current transmission rate. This can effectively control network congestion and improve the efficiency of real-time data transmission. It ensures high real-time and transmission quality of underwater multimedia data transmission.

In order to verify the stability of the system under different delay conditions in the presence of network jitter, experimental analysis was conducted using packet loss rate as the test indicator. The mathematical formula is as follows:


$$PLR=\frac{{ϒ}_{d}}{{ϒ}_{z}}$$ (6)

**Fig 9:**
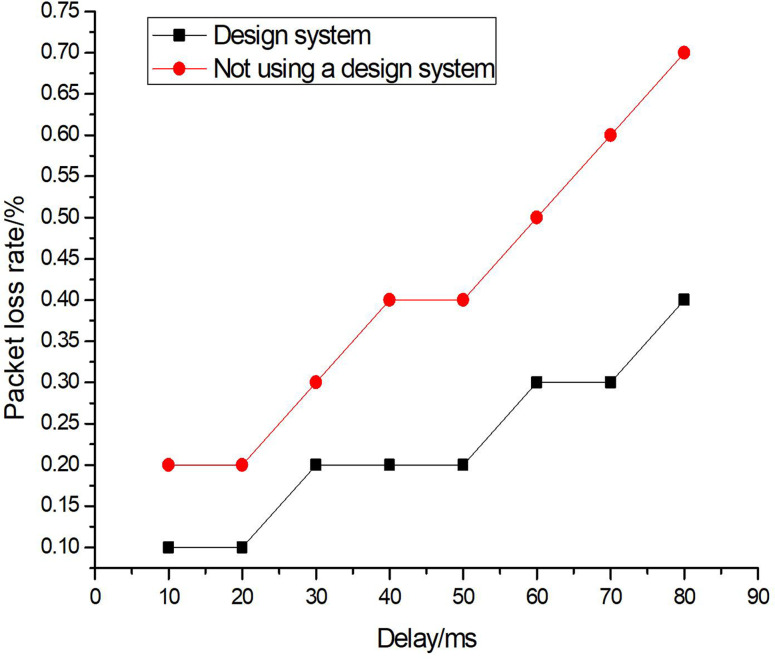
Analysis of packet loss rate test results.

Where, ${ϒ}_{d}$ represents the number of lost packets and ${ϒ}_{z}$ represents the total number of packets sent. The analysis results are shown in Fig 9.

According to the analysis of Fig 9, the packet loss rate of the system without application design is relatively high, reaching 0.7% at a delay of 80, while the packet loss rate of the system with application design remains below 0.5%, indicating that the system can still maintain good data transmission performance in a network jitter environment.

## 5 Conclusion

This paper mainly designs and implements a real-time transmission system for underwater panoramic cameras based on RTSP. The system achieves real-time collection, encoding, transmission, and reception of underwater panoramic video data through the collaborative work of the data acquisition layer, data encoding layer, RTSP server layer, and RTSP application layer. During the data transmission process, the system adopts the RTCP protocol for network congestion control to ensure the stability and reliability of data transmission. The experimental results show that the designed system can view the dynamics of fish in rivers in real time through the RTSP client, with clear transmission image quality and no lag phenomenon. In experiments where there is a shaking environment in the network, the packet loss rate of the designed system remained below 0.5%, far lower than the packet loss rate of the system without application. This indicates that using camera calibration methods to calibrate underwater panoramic cameras can improve the accuracy of image acquisition, and the application effect of the designed system is good.

Due to the complexity and uncertainty of underwater environments, the robustness of the system still needs to be further improved. In future research, it is necessary to further improve the imaging quality and data transmission stability of cameras in situations where underwater light changes dramatically or water currents are turbulent.
